# County-Level Radon and Incidence of Female Thyroid Cancer in Iowa, New Jersey, and Wisconsin, USA

**DOI:** 10.3390/toxics6010017

**Published:** 2018-03-16

**Authors:** Caroline Oakland, Jaymie R. Meliker

**Affiliations:** 1 Hampton Bays High School, Hampton Bays, NY 11946, USA; caroline.oakland@aol.com; 2 Program in Public Health, Department of Family, Population, and Preventive Medicine, Stony Brook University, Stony Brook, NY 11794, USA

**Keywords:** radon, thyroid, cancer, incidence, spatial, maps

## Abstract

Background: Few studies have investigated the association between radon and thyroid cancer despite the sensitivity of the thyroid gland to radiation. Our goal is to investigate the association between county-level radon and incidence of female thyroid cancer in the US States of Iowa, New Jersey, and Wisconsin. Methods: Thyroid cancer incidence data were provided by individual state cancer registries and span 1990–2013. Radon data come from a publicly available third-party database, AirChek, accessed in 2017. We tabulated the percent of radon above four picocuries per liter and the female thyroid cancer incidence rate in each county. Quantile maps were constructed, and an ordinary least-squares regression model was run using Geoda 1.10.0.8 (Chicago, IL, USA). Results: No association was observed between county-level radon and incidence of female thyroid cancer in any of the States: New Jersey (β = 0.06, *p* = 0.23); Iowa (β = −0.07, *p* = 0.07); or Wisconsin (β = −0.01, *p* = 0.78). A spatial regression model was considered, but the Moran’s I of the residuals from each of the models was not significant, so no spatial term was required. Discussion: In this county-level ecological study across three different States in the US, we did not find an association between elevated radon and thyroid cancer incidence in women. While this ecologic study reports null findings, due to the ecologic fallacy, individual-level studies of this association may still be warranted.

## 1. Introduction

Radon is a colorless, odorless, and tasteless gas produced by the radioactive decay of uranium in soil [1]. As a part of normal radioactive decay, radon becomes short-lived radioisotopes that can be inhaled by humans and layer the aero digestive tract [2]. Radon is often found in both basements and ground-level areas which leads to high-level exposure inside [3].

The United States (US) formed their radon measurement program in the 1950s. The US Environmental Protection Agency (USEPA) has promoted radon testing due to many areas within the US having elevated levels of radon [4]. Exposure to radon ≥4 picocuries per liter (pCi/L) is considered harmful to humans, mainly due to its association with lung cancer [5,6]. Radon is identified as a group I carcinogen and is the second leading cause of lung cancer in the United States [5]. As a result, many physicians promote in-house radon testing, which can reduce the risk of lung cancer [3]. In addition to lung cancer, radon may be associated with other diseases. Few studies have investigated the association between radon and thyroid cancer, despite the sensitivity of the thyroid gland to radiation.

Radiation exposure from atomic bombs impacted children’s thyroids; adolescents showed an increased risk of developing thyroid cancer in the five decades after atomic bomb exposure in Japan [7]. The thyroid gland is particularly susceptible to radioiodine exposure, although thyroid tumors can also be induced by other ionizing radiation, such as alpha particles from Pu^238^ [8]. Following Chernobyl, there was an increased incidence of thyroid cancer among children [9,10,11], with a suggestion of increased risk among adult Chernobyl liquidators as well [12], although evidence of risk among exposed adults is equivocal [13]. We hypothesize exposure to radon may be a risk factor for thyroid cancer. Thyroid cancer incidence has increased dramatically over the past two decades, particularly in women; it has become the 5th-most frequent cancer diagnosed in women, moving up nine places in twenty years [14,15]. The sudden rise in thyroid cancer incidence is likely partially due to an increase in thyroid cancer testing and technology with enhanced ability to investigate small non-palpable thyroid nodules [15]. However, thyroid cancer has still increased rapidly, which highlights the importance of identifying modifiable risk factors for thyroid cancer. While there is no evidence that radon exposure within homes is simultaneously increasing, susceptibility could change concurrently, perhaps due to epigenetic changes, which could lead to increased risk from exposures such as radon. To our knowledge, only two previous ecologic studies investigated radon and thyroid cancer incidence; one study in Ireland had only a single case [16], and a second study in Pennsylvania observed no association [17]. Given the sensitivity of the thyroid to radiation exposure, we felt this hypothesis deserved additional scrutiny.

Iowa, New Jersey, and Wisconsin are noted to have high radon levels—frequently above 4 pCi/L—and also had publicly available thyroid cancer incidence data (http://cancer-rates.info). This study evaluated the relationship between radon above 4pCi/L with the incidence rate of thyroid cancer in women in the counties of Iowa, New Jersey, and Wisconsin.

## 2. Materials and Methods

Publicly available databases were accessed for this study of county-level radon and county-level incidence of thyroid cancer. The rates of thyroid cancer were downloaded from http://cancer-rates.info for the years 1990–2013; data were obtained from the cancer registries in each state. Thyroid cancer cases were limited to females of all ethnicities, and the 2000 US Standard Population was used to estimate the population at risk. Data downloaded included the population at risk, number of cases, and the age-adjusted rate of thyroid cancer for each county in the three states. Because of small numbers of cases in some counties, 12 of Iowa’s 99 counties were missing data, as were 4 of Wisconsin’s 72 counties.

For radon data, a third-party database was used called AirChek (http://state-radon.info). Individual National Environmental Health Association-certified radon specialists measured radon with kits that were collected from individual homes. The number of samples analyzed in each county has not been reported, but millions of samples have been analyzed across the US. These data were captured as the percent of radon above 4pCi/L for each county. 

A Pearson correlation coefficient was calculated between the age-adjusted rate and percent of radon above 4pCi/L in each county. Quartile maps were constructed for both the thyroid age-adjusted rates and the percent of radon above 4pCi/L using GeoDa 1.10.0.8 (Chicago, IL, USA). In GeoDa, an ordinary least-squares regression was calculated between the independent variable, percent of radon above 4pCi/L, and dependent variable, age-adjusted incidence rate. As a regression diagnostic, spatial autocorrelation in the residuals was assessed by calculating a Moran’s I coefficient using Queen’s nearest neighbors with an order of one.

## 3. Results

New Jersey had the most female thyroid cases reported (N = 16,906), followed by Wisconsin (N = 7250), and Iowa (N = 4236). Correlation coefficients between percent of radon over 4pCi/ L and age-adjusted thyroid cancer incidence rate were calculated for each of the three states, and overall, and all correlations showed no association (Table 1).

**Figure 1:**
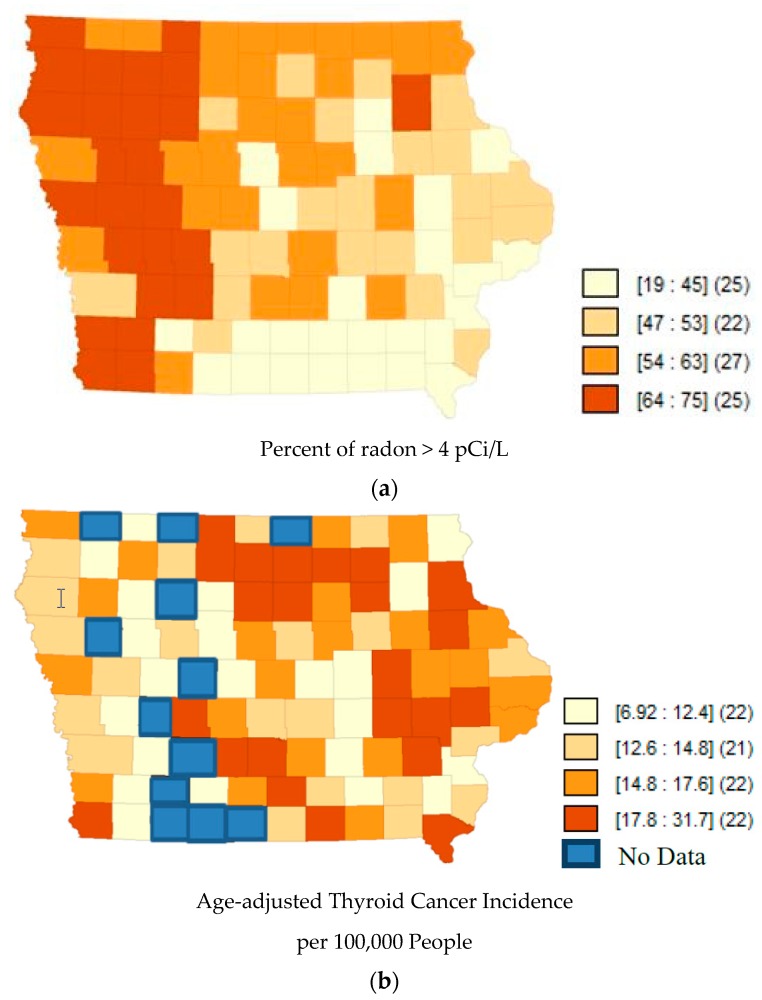
(**a**) Map of Quartiles of Radon Concentrations in Iowa counties. Darker shades represent a higher percent of radon above 4 pCi/L. The numbers inside the brackets represent the range of percentages in that shade. The numbers inside the parentheses represent the number of counties in that quantile. Data accessed from AirChek, 2017. (**b**) Map of Quartiles of Age-adjusted Thyroid Cancer Incidence (1990–2013) in Iowa counties. Darker shades of orange represent higher rates of thyroid cancer incidence. The blue color represents the counties that lacked data from the public database. The numbers inside the brackets represent the range of incidence rates per 100,000 in that shade. The numbers inside the parentheses represent the number of counties in that quantile.

**Figure 2:**
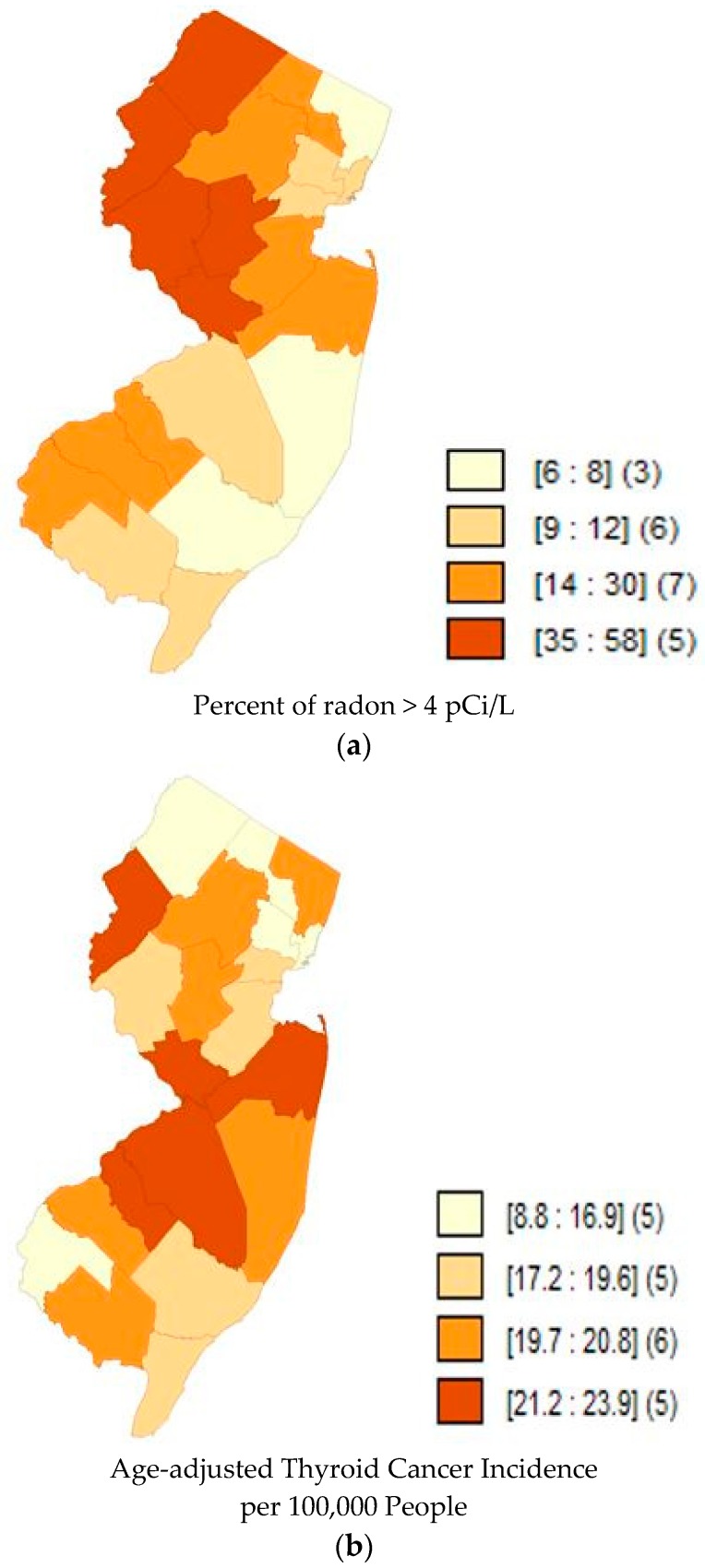
(**a**) Map of Quartiles of Radon Concentrations in New Jersey counties. Darker shades represent a higher percent of radon above 4 pCi/L. The numbers inside the brackets represent the range of percentages in that shade. The numbers inside the parentheses represent the number of counties in that quantile. Data accessed from AirChek, 2017. (**b**) Map of Quartiles of Age-adjusted Thyroid Cancer Incidence (1990–2013) in New Jersey counties. Darker shades of orange represent higher rates of thyroid cancer incidence. The numbers inside the brackets represent the range of incidence rates per 100,000 in that shade. The numbers inside the parentheses represent the number of counties in that quantile.

**Figure 3:**
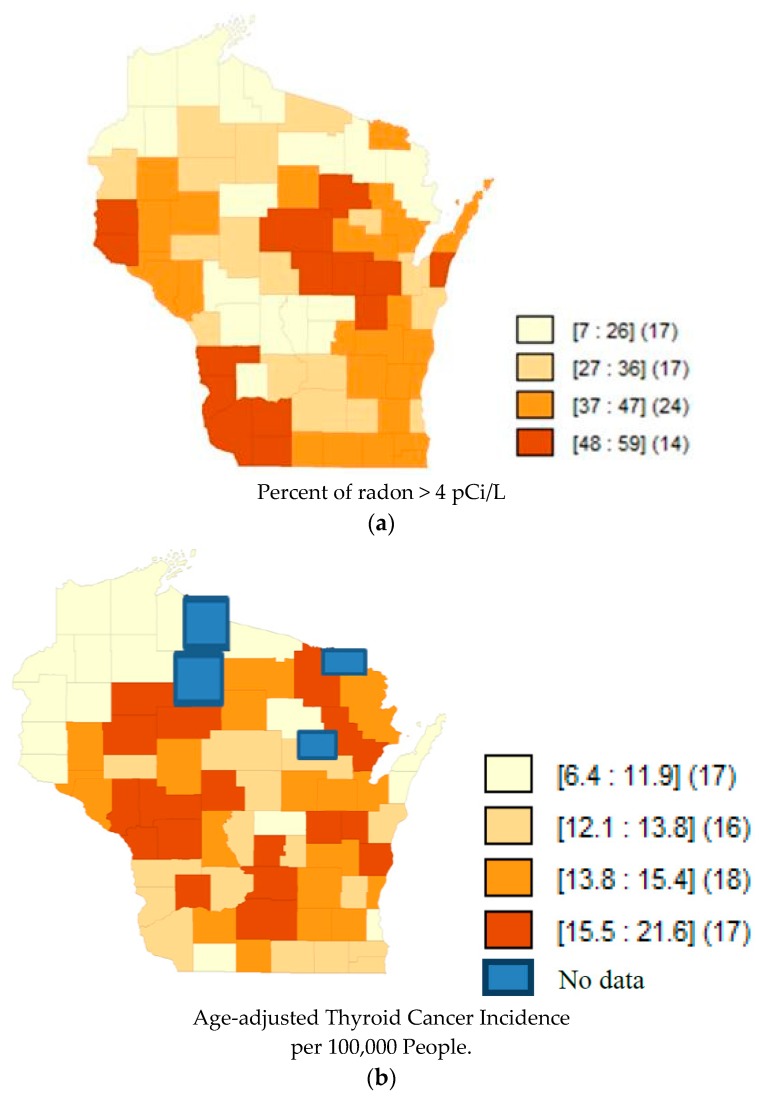
(**a**) Map of Quartiles of Radon Concentrations in Wisconsin counties. Darker shades represent a higher percent of radon above 4 pCi/L. The numbers inside the brackets represent the range of percentages in that shade. The numbers inside the parentheses represent the number of counties in that quantile. Data accessed from AirChek, 2017. (**b**) Map of Quartiles of Age-adjusted Thyroid Cancer Incidence (1990–2013) in Wisconsin counties. Darker shades of orange represent higher rates of thyroid cancer incidence. The blue color represents the counties that lacked data from the public database. The numbers inside the brackets represent the range of incidence rates per 100,000 in that shade. The numbers inside the parentheses represent the number of counties in that quantile.

Quartile maps (Figure 1, Figure 2 and Figure 3) showed no visual association between higher radon and higher incidence rates; and the regression models confirmed that there was no association (Table 2). Moran’s I of the residuals was not significant for any of the models, indicating that spatial autocorrelation was not a concern.

## 4. Discussion

In this county-level ecological study across three different States in the US, we did not find an association between elevated radon and thyroid cancer incidence in women. We hypothesized that radon may be a risk factor for thyroid cancer because of the thyroid gland’s sensitivity to radiation exposures [7,8,9,10]. It is now understood that radon itself becomes an inert gas relatively quickly, but its decay products, including radioisotopes of polonium, lead, and bismuth, are believed to pose the risk [18]. While radioactive iodine is most clearly linked with thyroid cancer because of the thyroid gland’s affinity for iodine, thyroid tumors have also been induced by exposure to other sources of ionizing radiation, including plutonium [8]. Therefore, the hypothesis that radon, one of the most common sources of radiation exposure in the population, is associated with thyroid cancer merits focused interrogation.

Increased risk of radiation from atomic bombs and nuclear explosions (e.g., Chernobyl) has been documented among those exposed as children [7,8,9,10], indicating exposures during childhood may be a critical time window. Adult radiation exposures, however, were not associated with thyroid cancer in a pooled analysis [19], but in more recent analyses of atomic bomb survivors [20] and Chernobyl liquidators [12], showed a slightly increased risk. Given the documented long latency period between low-level radiation exposure (as low as 0.09 Gy over a lifetime) and thyroid cancer incidence [19], an ecologic study may not be robust enough to identify this association.

To our knowledge, only two previous studies have investigated thyroid cancer in relation to radon exposure, and those ecologic studies in Iceland and in Pennsylvania were also conducted using large area exposure averages, and they failed to detect an association [16,17]. Our study expands on those studies to include three states, with more than 28,000 total cases, and greater opportunity to examine consistency across states.

The limitations of this study largely revolve around the ecologic nature of the investigation. We did not have individual-level data on radon exposure or thyroid cancer, nor were we able to account for early life exposure. While we show that county-level age-adjusted female thyroid cancer incidence rates were not associated with counties with higher rates of radon >4 pCi/L, we do not know whether individuals lived in these counties during earlier periods of their life, nor whether they were actually exposed to elevated levels, be it in their own homes or at any other places such as work or school. Therefore, while this ecologic study reports null findings, we cannot refute the possibility that individual thyroid cancer cases may have been exposed to higher levels of radon.

## 5. Conclusions

We did not find an association between county-level radon and age-adjusted incidence rates of female thyroid cancer in Iowa, New Jersey, and Wisconsin. More detailed individual-level research with estimates of exposures during early childhood may be needed to determine whether or not we can discard the hypothesis that radon causes thyroid cancer.

## Figures and Tables

**Table 1:** Correlation coefficient between percent of samples >4 pCi/L and age-adjusted thyroid cancer incidence rates in county-level data from three US States. Cancer incidence 1990–2013.

States	Correlation Coefficient	*p*-Value
Iowa	−0.19	0.07
New Jersey	0.28	0.23
Wisconsin	−0.03	0.78
All 3 States	−0.12	0.12

**Table 2:** Beta coefficients from ordinary least-squares regression of percent of radon above 4 pCi/L on age-adjusted thyroid cancer incidence rate in county-level data from 3 US States. Cancer incidence 1990–2013.

States	Beta Coefficient	*p*-Value
Iowa	−0.07	0.07
New Jersey	0.06	0.23
Wisconsin	−0.01	0.78
